# Membrane protein dynamics: limited lipid control

**DOI:** 10.1186/1757-5036-2-1

**Published:** 2009-02-06

**Authors:** Balázs Szalontai

**Affiliations:** 1 Institute of Biophysics, Biological Research Center, Hungarian Academy of Sciences, H-6701 Szeged, Temesvári krt. 62, P.O.B. 521, Hungary

## Abstract

Correlation of lipid disorder with membrane protein dynamics has been studied with infrared spectroscopy, by combining data characterizing lipid phase, protein structure and, via hydrogen-deuterium (H/D) exchange, protein dynamics. The key element was a new measuring scheme, by which the combined effects of time and temperature on the H/D exchange could be separated. Cyanobacterial and plant thylakoid membranes, mammalian mitochondria membranes, and for comparison, lysozyme were investigated. In dissolved lysozyme, as a function of temperature, H/D exchange involved only reversible movements (the secondary structure did not change considerably); heat-denaturing was a separate event at much higher temperature. Around the low-temperature functioning limit of the biomembranes, lipids affected protein dynamics since changes in fatty acyl chain disorders and H/D exchange exhibited certain correlation. H/D exchange remained low in all membranes over physiological temperatures. Around the high-temperature functioning limit of the membranes, the exchange rates became higher. When temperature was further increased, H/D exchange rates went over a maximum and afterwards decreased (due to full H/D exchange and/or protein denaturing). Maximal H/D exchange rate temperatures correlated neither with the disorder nor with the unsaturation of lipids. In membrane proteins, in contrast to lysozyme, the onsets of sizable H/D exchange rates were the onsets of irreversible denaturing as well. Seemingly, at temperatures where protein self-dynamics allows large-scale H/D exchange, lipid-protein coupling is so weak that proteins prefer aggregating to limit the exposure of their hydrophobic surface regions to water. In all membranes studied, dynamics seemed to be governed by lipids around the low-temperature limit, and by proteins around the high-temperature limit of membrane functionality.

**PACS codes**: 87.14.ep, 87.14.cc, 87.16.D

## 1. Background

Maintaining the barrier properties and the functioning (energy production, signal transduction, material transport etc.) of a biological membrane, a given membrane dynamics is required. This dynamics is adapted to the physiological conditions (e.g. temperature, light in photosynthetic organisms, different stresses) of the given membrane. Concerning barrier properties, the dynamics of membrane lipids and lipid-protein interactions are thought to be more important. The biological functioning is assured by the dynamics of the membrane proteins, which may depend also on lipid-protein interactions. The balance between lipid- and protein-dynamics and the role of lipid-protein interaction in maintaining membrane functionality is still not fully understood.

It has been shown previously in a series of papers, mostly for photosynthetic organisms, that at the physiological low temperature limit and among cold-stress conditions, the physical state of the lipids and the capacity of the living organism to regulate it are very important factors in the survival and adaptation [1-5]. As regards signal transduction, changes of lipid composition in rat ventricular myocyte cell membranes were shown to affect receptor-mediated processes [6]. Around the high temperature physiological limit and among heat-stress conditions, a histidine kinase has been identified as a source of signalling for increased thermo tolerance [7]. In another series of papers, the membrane protecting function of heat-shock proteins [8], and the existence of "hyperfluid" lipid domains in the membranes (on the analogy of the effects of fluidising agents) have been proposed as sources of the heat-shock alarm signal [9,10]. We have shown earlier that around or slightly above the physiological high temperature limit of the living organism a rather steep membrane-protein denaturing appears [11], while the lipid disorder in biological membranes at high temperatures is a very smoothly changing feature [11,12]. From a teleological point of view, a feature, which is changing steeply in the region of interest, would be better suited as triggering signal. Summarizing the literature data, it seems that there is consensus that lipid composition and dynamics have the primary role in low-temperature adaptation and cold stresses. At the high temperature end, however, it is not clear, whether proteins or lipids, or their specific interactions are the primary actors in maintaining membrane structure and/or stress signalling. In most of those studies, there was no specific attention paid to the dynamic aspects of the observed phenomena.

Here, we make an effort to answer some of these open questions by addressing the basic problem, whether lipids, or proteins are the primary actors in maintaining membrane dynamics. For this end, we use specifically designed experimental approach by exploiting H/D exchange, which, taken into account its capacity of measuring molecular dynamics directly, seemed to be the choice for such a study.

**Figure 1:**
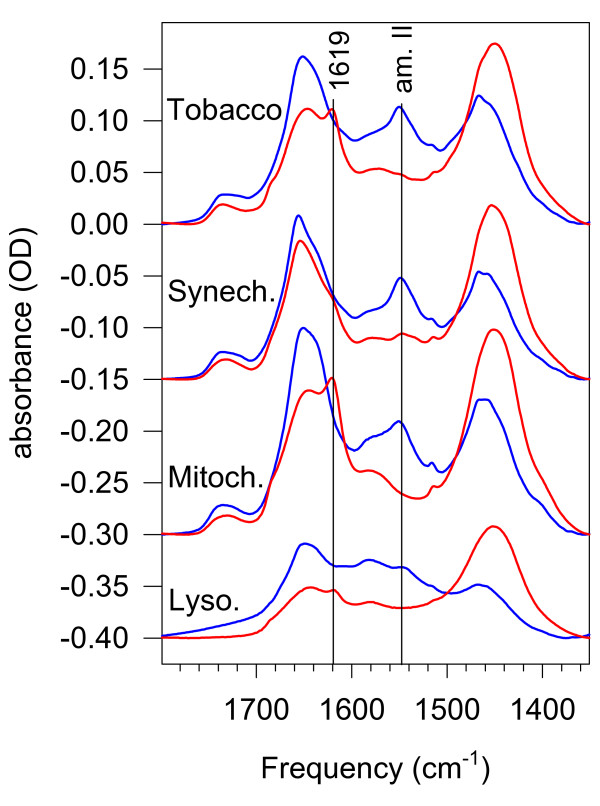
**Amide I-II-II' regions of the studied membranes and lysozyme at around 5°C and 80°C**. Note the different extent of amide II diminishing and protein denaturing (the band at 1619 cm^-1^) in the different samples. Blue curve – 5°C, red curve – 80°C. Tobacco – tobacco thylakoid; Synech. – thylakoid membrane of a cyanobacterium, *Synechococcus PCC7942*; Mitoch. – rat liver mitochondrial membrane; Lyso. – water-dissolved lysozyme enzyme. Spectra were displaced for clarity.

Hydrogen-deuterium exchange has long been utilized to study protein structure [13-15]. The method is based on the fact that H atoms bound to N or O are exchangeable with the protons or deuterons of the surrounding H_2_O or D_2_O. Since the contribution of the N-H deformation to the amide II band at around 1550 cm^-1 ^is large, large isotopic shift occurs upon replacing N-H groups by N-D ones when a protein is dissolved in D_2_O. Therefore, as H/D exchange progresses, parallel to the disappearance of the 1550 cm^-1 ^band, and a new band appears at around 1450 cm^-1 ^named amide II' (Figure 1). This change of the infrared spectrum is utilized to follow the kinetics of the H/D exchange. Since the accessibility of the external medium to different protein domains is depending on fluctuations of the protein structure, the rate of the H/D exchange is a true and direct measure of protein dynamics.

In water-dissolved proteins, the self-dynamics of the protein is determining the access of the external medium to the interior of the molecule. Using this approach, a wealth of information has been obtained on the dynamics of proteins by classifying the peptide protons of the proteins according to their H/D exchange rates [16-18].

The situation is more complex and markedly different in the case of membrane proteins [19]. Here, there are extra-membrane regions of the protein surface, which are accessible from the surrounding water phase, and there are hydrophobic surface regions, which are in contact with the lipid fatty acyl chains in the interior of the membranes. Through this latter phase, the movements of the H_2_O/D_2_O molecules are restricted, and in addition, the extent of this restriction may depend on the physical state of the surrounding lipid matrix. The effectiveness of this membrane-related limitation on the H/D exchange in membrane proteins is shown by the fact that biological membranes have to be heated up to about 40–50°C to exhibit observable H/D exchange rates (see Figure Three in [20]).

So far not much attention has been paid to the role of the lipid physical state in the H/D exchange of membrane proteins, in spite of the fact that the structure of the lipid double layers is well understood [21]. Formation of lipid domains in model and biological membranes has been extensively studied with vibrational spectroscopy [22]. The most frequently utilized infrared spectroscopic parameter in these systems is the frequency of the symmetric CH_2 _stretching mode near 2850 cm^-1^. This frequency has been shown to increase by 2–5 cm^-1 ^upon gel → liquid crystalline phase transition. The temperature dependence of this frequency shift is a sensitive measure of lipid conformations, and of lipid-protein interactions (for a review on ν_sym_CH_2 _in lipids, see ref. [23]). Fine details of lipid fatty acyl chain conformations and how they are related to the membrane structure have also been revealed by CH_2 _wagging progression [24]. As regards biological membranes, the adaptation to growth temperatures and the corresponding changes in lipid disorder could also be followed by FTIR spectroscopy [12,25].

For the first time, we have investigated systematically this problem, in the hope that through the study of the dynamics, the heat-denaturing of membrane proteins, and by taking into account the effect of the lipid matrix as well, we can learn more about the organization of biological membranes, and about the role of lipid-protein interaction in it. In the present study, the following features will be examined and the obtained data compared: (i) changes in the amide I-II region due to H/D exchange, (ii) changes in the disorder of the lipid fatty acyl chains in the membrane via looking at the temperature dependence of the frequency of the ν_sym_CH_2 _band, and (iii) the evolution of a band in the amide I region at around 1619 cm^-1^, which is diagnostic for protein heat-denaturing [26].

For this purpose, three different biological membranes and a water-dissolved protein were chosen: (i) thylakoid membranes of tobacco, a higher plant, having high level of lipid unsaturation [27] and high protein-to-lipid ratio; (ii) thylakoid membranes from *Synechococcus *PCC 7942, a cyanobacterium with low level of lipid unsaturation [28] and low protein-to-lipid ratio; (iii) rat liver mitochondria with high level of lipid unsaturation [29], and low protein-to-lipid ratio; and for control, (iv) water-dissolved lysozyme. With that selection, we wanted to address the problem of the possible roles of protein-to-lipid ratios and lipid unsaturation as well in the membrane dynamics and lipid-protein interaction.

## 2. Methods

### 2.1. Samples

Tobacco thylakoid membranes were isolated and prepared for infrared measurements as described previously [11]. Thylakoid membranes of *Synechococcus *PCC 7942, a mezophyl cyanobacterium grown at 42°C were a gift from Dr. S. Allakverdiev [30]. Rat liver mitochondrial membranes were a gift from Dr. I. Horváth [29]. Lysozyme was purchased from Sigma and was used at a concentration of 10 mg.mL^-1^. All membrane suspensions were stored at -70°C before use, and then re-suspended in D_2_O-based PBS buffer (10 mM, pD 6.6), centrifuged down and washed once with the same buffer.

### 2.2. Infrared measurements

FTIR spectra were recorded on a Philips PU9800, or on a Bruker IFS66 Fourier transform infrared spectrometer, averaging 128 scans at 2 cm^-1 ^spectral resolution by using a sample shuttle. At each temperature, a background and a sample spectrum was recorded. A water-thermostated cell holder controlled the temperature of the sample. The accuracy of the temperature setting was about 0.1°C. Spectrum manipulations were carried out with the SPSERV© software of Dr. Cs. Bagyinka (Biological Research Center, Szeged, Hungary). Samples were prepared from frozen stocks, which were brought to 4°C. The amount required for one measurement was taken out (≈ 100 μL) diluted with D_2_O-based buffer (≈ 1 mL), centrifuged down, re-suspended in 1 mL D_2_O-based buffer, centrifuged again and the pellet was used for the infrared measurements, immediately. The pellet (about 20 μL) was placed between CaF_2 _windows separated by an aluminium spacer (15 μm). The whole preparation process took about 30 minutes until the sample was in place in the spectrometer. An additional 30-minute waiting was needed before the actual start of the FTIR measurements to decrease the water vapour level in the spectrometer.

### 2.3. H/D exchange protocol

A new measuring protocol was developed for studying H/D exchange in biological membranes. At each temperature, two infrared absorption spectra were recorded with 7 min time interval (e.g. S(1), S(2)) and then the temperature was raised by 3°C. (This value is only an indicator of the temperature steps, the actual temperature of the sample was measured by an independent thermometer at the moment when the recording of an infrared spectrum started. These temperature values could deviate somewhat from the equidistance, due to the varying loss with increasing temperatures. We accepted this, since equidistant timing of the measured spectra had priority. For all data evaluation the concrete sample temperatures were used.) Another 7 min was left to reach equilibrium at the new temperature, at which again two spectra were recorded (e.g. S(3), S(4)). This cycle was repeated between 5–80°C. From the S(i)_i = 1, n _measured spectra, a series of difference spectra were calculated each time by subtracting the previous spectrum from the following one. Thus, a new D(i) = S(i+1)-S(i)_i = 1, n-1 _series was obtained. Its odd elements represent differences between spectra recorded at the same temperature (e.g. D(1) = S(2)-S(1)). These will be called isotherm difference spectra (ISO). The even elements of the D(i) series represent differences between infrared absorption spectra, which were recorded after and before the temperature step (e.g. D(2) = S(3)-S(2)). These will be called temperature difference spectra (ΔT).

### 2.4. Evaluation of the infrared difference spectra

In the amide I region, the intensity of the band (around 1619 cm^-1^) characteristic of protein denaturing was determined by integrating it between 1634–1596 cm^-1^. The intensity and the frequency of the disappearing fraction of the amide II band (due to the H/D exchange) were determined by fitting the difference spectra with a Lorentzian curve between 1586–1502 cm^-1^. The evaluation of the spectra was an iterative process; on the first trial, the very weak difference spectra gave nonsense results. With increasing temperature, at a certain point, changes became big enough for a reasonable fit (i.e. the fitting program (SPSERV) could find by itself a solution). From here on, the fits were done by leaving free all parameters (intensity, frequency, bandwidth) of the Lorentzian curve Then, the previously nonsense fits of the first, weak difference spectra were replaced with fits where the frequencies were fixed at values obtained from the closest spectrum with reasonable fit (just to provide acceptable starting values for the later changes). In the spectra fitted with free parameters, the maximal error in the frequency determination was less than 2 cm^-1^; in the intensity it was less than 15%.

## 3. Results

The total changes in the amide I-II region upon time and heating from 5 to 80°C for all the studied systems are depicted in Figure 1. It can be seen that at the beginning of each experiment sizable amide II band at around 1550 cm^-1 ^was present. Most of it disappeared upon increasing the temperature, but the H/D exchange was not complete. We believe that there was no major H/D exchange before the start of the measurements, because up to room temperatures and a bit above the exchange rates remained very low (*vide infra*).

### 3.1. Membrane with high level of lipid unsaturation and high protein-to-lipid ratio: Tobacco thylakoid

#### 3.1.1. H/D exchange in tobacco thylakoid membranes

**Figure 2:**
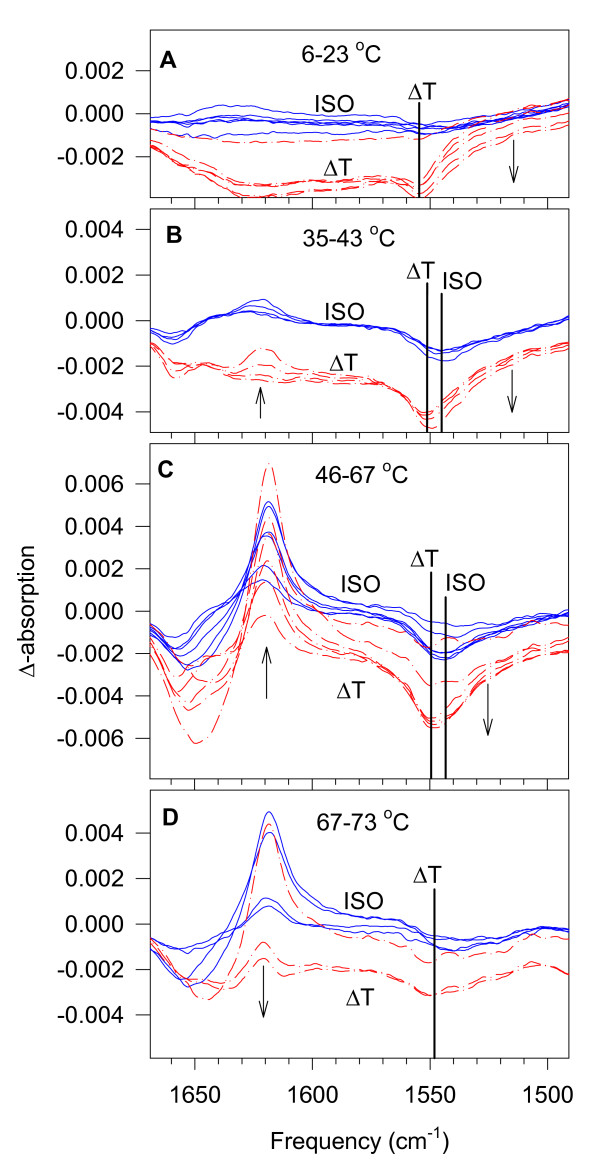
**Difference spectra for tobacco thylakoid membranes obtained by calculating D(i) = S(i+1) – S(i) from the series of measured S(i) spectra**. Continuous blue curves indicate ISO difference spectra, where the temperature of the two spectra from which these difference spectra were calculated was the same. Red dash-dot curves indicate ΔT difference spectra, where there was about 3°C difference between the spectra from which these difference spectra were calculated. Arrows show the directions of the changes at the given spectrum region upon increasing temperature. Thicker lines show the different minima of the disappearing amide II band in the ISO and the ΔT spectra. The difference spectra of the studied temperature range (6–73°C) are divided into four panels according to their tendencies to change. The intensity scaling in the four panels is the same.

The series of isotherm (ISO) and temperature difference (ΔT) difference spectra are shown in Figure 2. The spectra are grouped into four temperature ranges according to their characteristic features.

Between 6–23°C, the first negative changes appeared in the amide II region of the ΔT spectra indicating the beginning of H/D exchange (Figure 2A).

At around 35°C, the diminution of the amide II band appeared also in the ISO spectra (Figure 2B). In the amide I region, the evolution of a band at around 1619 cm^-1 ^started first and more strongly in the ΔT spectra, then in the ISO spectra. This band was related to intermolecular β-structures upon heat denaturing of proteins [26]. Here, this is probably not the case from the very beginning. In the first difference spectra when this band is weak yet, it is between 1630–1620 cm^-1^. These frequencies are characteristic of intra-molecular β-structures (Figure 2B).

From about 46°C on, the H/D exchange accelerated (Figure 2C). Both the ISO and ΔT spectra exhibited their largest amplitudes here. The amide I region became dominated by the 1619 cm^-1 ^band of protein denaturing. The frequency of the α-helix-related negative band, which is apparently the source of the increase of the inter-molecular β-structures, has downshifted from 1655 to about 1650 cm^-1^, which might be due to H/D exchange parallel to the structural change.

There was, however, a temperature, where the rate of the H/D exchange started to decrease. In the case of tobacco, at the present timing of the experiments it was at around 67°C (Figure 2D). It has to be kept in mind that H/D exchange is a 'historic' process. What has happened cannot be repeated. As H/D exchange goes on, its conditions are changing continuously. Not the same protons and not from the same places are exchanged as time passes. In this process, therefore, the temperature and the timing are important parameters. For our purposes, we found the time difference of 7 min between the subsequent spectra convenient. There were still enough protons to be exchanged when reaching the temperature range of protein denaturing. In spite of the differently situated H atoms involved, correlation among changes in the amide II, amide I, and of the C-H stretching regions reflects real interdependence among H/D exchange, protein structure and lipid phase. Here, the diminishing of the amide II band slowed down while protein denaturing continued, the band at 1619 cm^-1 ^remained intensive in the difference spectra, but later that band also weakened. The reason can be a more limited access to still non-exchanged protons in the interior of the aggregated denatured proteins. The complete heat-denaturing of these membrane proteins can be expected at higher temperatures (above 80°C), here we focused on the changes appearing around the high-temperature functioning limit of the membranes studied.

#### 3.1.2. Lipid disorder in tobacco thylakoid membranes

**Figure 3:**
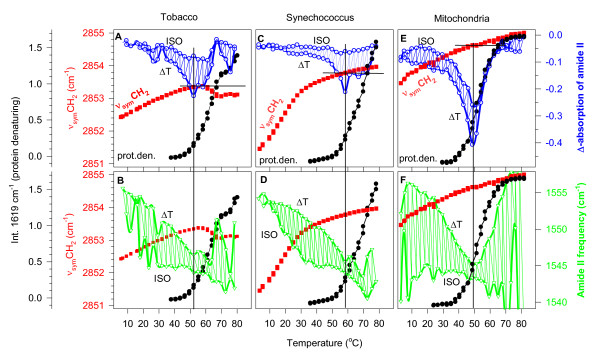
**The temperature dependences of protein denaturing, H/D exchange and lipid fatty acyl chain disorder in different biological membranes**. Comparison of H/D exchange rates (open circle) (panels A, C, and E), and the characteristic frequencies (open triangle) (panels B, D, and F) of the disappearing amide II band with the lipid disorder (open square) (ν_sym_CH_2_) and with protein heat-denaturing (black circle) (prot. den.) in tobacco (panels A, B), in *Synechococcus *(panels C, D) thylakoids, and in mitochondrial membranes (panels E, F). Note that the scale of the disappearing amide II intensity changes is negative, thus lower values mean higher exchange rates. Vertical lines indicate the temperatures of the maximal H/D exchange rates; these temperatures do not correlate with the level of the corresponding lipid disorder (ν_sym_CH_2 _points) indicated with horizontal lines in panels A, C and E. For the meaning of ISO and ΔT, see Methods – *H/D exchange protocol*.

**Figure 4:**
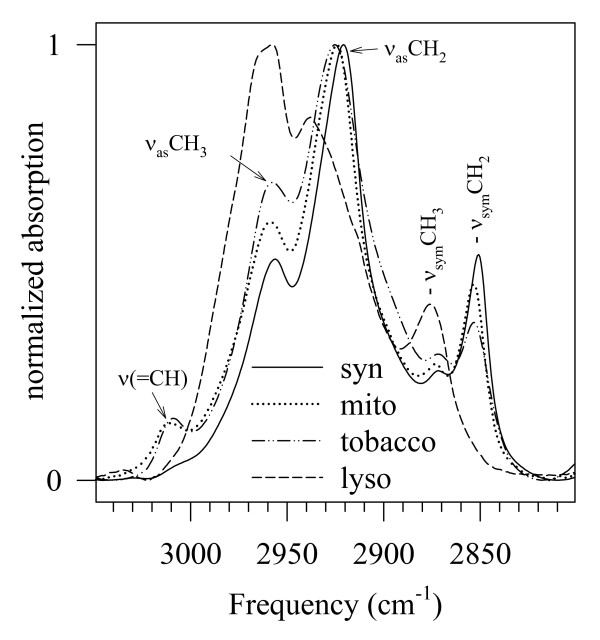
**The C-H stretching regions of Synechocystis thylakoids, rat liver mitochondrial membrane, tobacco thylakoids, and lysozyme**. Higher relative intensity of the ν_as_CH_3 _means higher protein-to-lipid ratio, more intensive ν (= CH) bands higher levels of lipid unsaturation in a membrane (For explanation, see the text.)

Lipid disorder in tobacco thylakoid membranes as a function of the temperature was determined from the shift of the ν_sym_CH_2 _band [21,31]. The C-H stretching regions of the investigated membranes are given in Figure 4. For the determination of the ν_sym_CH_2 _frequency shift, the C-H stretching region (3050–2800 cm^-1^) was fitted with Lorentzian component bands as earlier [12]. The frequency of the component band fitted to the ν_sym_CH_2 _band is plotted versus temperature in Figure 3A. The ν_sym_CH_2 _frequencies start at a high value (~2852.4 cm^-1^) at 5°C, and exhibit a modest, only about 1 cm^-1 ^increment up to 60°C. Then there is a slight decrease, and around 70–75°C the ν_sym_CH_2 _frequency levels off. This phenomenon is discussed in details in ref. [11]. These values are in agreement with the highly unsaturated fatty acid composition of the tobacco thylakoid membrane [27]. In this respect, see also the high relative intensity of the band assigned to = CH stretching at around 3011 cm^-1 ^for tobacco in Figure 4. The relative intensity of the band at around 2958 cm^-1 ^is indicative of the relative amount of CH_3 _groups in the membranes. Since the ratio of the CH_3_/CH_2 _groups is much higher in proteins (about 1:2) than in lipids (1:15,17,19) the higher relative intensity of the 2958 cm^-1 ^band is reflecting a higher protein-to-lipid ratio. (For comparison, see the relative intensity of this band in lysozyme in Figure 4.)

#### 3.1.3. Heat-induced protein denaturing in tobacco thylakoids

While both the ISO and the ΔT spectra show the evolution of the denaturing-related 1619 cm^-1 ^band higher temperatures, due to their 'derivative' nature, they are not suitable to determine the real extent of protein denaturing as a function of temperature. For this, we have used the original set of the spectra, S(i)_i = 1, n_. First, the amide I regions (1700–1600 cm^-1^) were normalized by dividing each data point by the average absorption in the amide I region. Then, a new series of difference spectra was created by subtracting the first spectrum from the later ones as earlier [11]. As witnessed by the emerging intensity of the 1619 cm^-1 ^band in Figure 3A, protein denaturing starts at around 48°C, it is the steepest at around 60°C, it has a plateau between 67–73°C, then denaturing continues to progress. The steepest region and the plateau correlate with the maximum and the levelling-off regions of the ν_sym_CH_2 _frequencies, respectively.

### 3.2. Membrane with low level of lipid unsaturation and lower protein-to-lipid ratio: Synechococcus PCC 7942 thylakoid

#### 3.2.1. H/D exchange in Synechococcus PCC 7942 thylakoid membranes

In the same experiments, as above for tobacco, we have obtained similar ISO and ΔT difference spectra for *Synechococcus *(not shown). As functions of the temperature, the rates of the disappearance of the amide II band are shown in Figure 3C, the frequencies oscillating between the ISO and ΔT spectra in Figures 3D.

#### 3.2.2. Lipid phase transition in Synechococcus PCC 7942 thylakoid membranes

The double-bond content in *Synechococcus *is much lower than in tobacco (it contains only mono-unsaturated fatty acids [28], see also in Figure 4 the lower relative intensity of the band assigned to = CH stretch at around 3011 cm^-1^). Thus, in *Synechococcus *almost a full gel-to-liquid crystalline phase transition can be detected when plotting ν_sym_CH_2 _frequencies versus temperature (Figure 3C). This ν_sym_CH_2 _thermotropic response curve contains all the measured S(i)_i = 1, n _spectra.

#### 3.2.3. Heat-induced protein denaturing in Synechococcus PCC 7942 thylakoids

The temperature dependence of the intensity of the 1619 cm^-1 ^marker band is plotted in Figure 3C. The onset temperature of protein denaturing here is at around 53°C, about 5°C higher than it was in tobacco.

### 3.3. Membrane with high level of lipid unsaturation and with lower protein-to-lipid ratio: Rat liver mitochondrial membrane

The protein-to-lipid ratio in the mitochondrial membrane is lower as compared to that of tobacco, but somewhat higher than that in *Synechococcus*. This can be seen in Figure 4 from the higher relative intensity of the band at around 2958 cm^-1^.

#### 3.3.1 H/D exchange in mitochondrial membranes

The total H/D exchange in the amide I-II region between 5–80°C is shown in Figure 1. Without presenting the corresponding ISO and ΔT difference spectra, which were very similar in character to those depicted for tobacco in Figure 2, we show the temperature dependence of the H/D exchange rate by the amide II intensity differences (Figure 3E), and by the frequencies of the disappearing amide II band (Figure 3F). Here also, like in the thylakoid membranes, ΔT spectra are always more intensive than the ISO spectra. The rate of the H/D exchange dramatically increases when denaturing of the proteins starts and diminishes as it is completed. The amide II frequencies of the mitochondrial ΔT spectra exhibit similar (practically linear) temperature dependence as in tobacco and *Synechococcus *thylakoids until roughly the midpoint temperature of protein denaturing. The amide II frequencies in the ISO spectra, however, were constant all over the investigated temperature range (Figure 3F). One should not be mislead by the large oscillations in the ISO amide II frequencies at very low and very high temperatures (neither here nor in tobacco); the intensities of the ISO spectra were very small in these regions (see Figure 3E), and therefore the determination of the amide II minima was rather uncertain.

#### 3.3.2. Lipid disorder in mitochondrial membranes

Lipid disorder in mitochondrial membranes is high, exhibiting an elevated ν_sym_CH_2 _starting value (~2853.4 cm^-1^), which is increasing by about 1 cm^-1 ^between 5 and 80°C (Figure 3E). This high degree of disorder agrees well with the high amount of C = C double bonds in the fatty acyl chains of the mitochondrial membrane evidenced by the high relative intensity of the ν (= CH) band in Figure 4. According to the similar relative intensity of this band, the level of lipid unsaturation in mitochondria should be expected roughly as high as in the tobacco thylakoid membrane. According to biochemical data, however, the double-bond index of the mitochondrial membrane is 1.73 as calculated from ref. [29]; for tobacco it is 2.31 on the basis of ref. [27]. It can be also seen from the ν_sym_CH_2 _frequency values that the double bond content is not the only parameter, which in membranes determines the lipid disorder, at least not that disorder we are measuring with ν_sym_CH_2_. In tobacco, at higher double bond index the ν_sym_CH_2 _frequencies were about 1 cm^-1 ^lower than in the mitochondrial membranes. Evidently, the lipid composition and the protein-to-lipid ratio of a membrane also have to be taken into account.

#### 3.3.3. Protein heat-denaturing in mitochondrial membranes

Protein heat-denaturing in mitochondrial membranes starts at around 40–42°C, and it is completed at around 65–70°C (Figure 3E). This is a major difference as compared to the behaviour of proteins in thylakoid membranes, where protein denaturing was far from being complete even at 80°C as indicated by the steep increase of the intensity of the 1620 cm^-1 ^band there (Figures 3A and 3C).

### 3.4. H/D exchange in a dissolved protein: lysozyme

**Figure 5:**
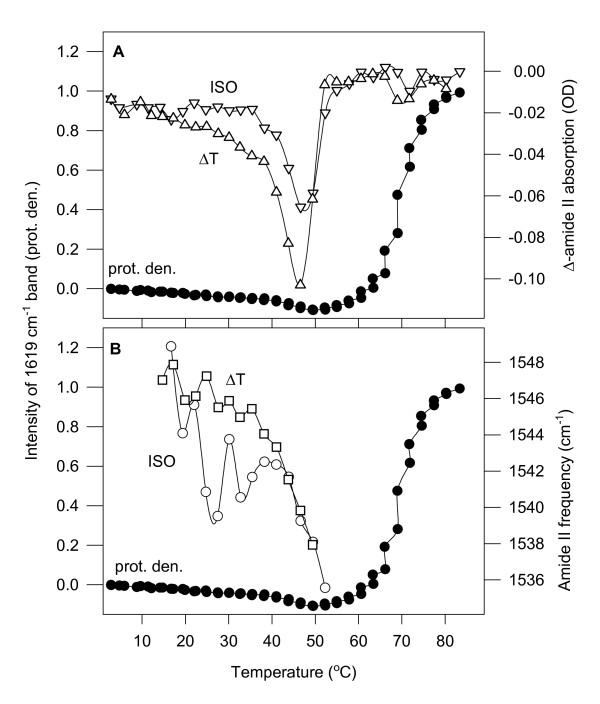
**H/D exchange in D_2_O-dissolved lysozyme**. Note that there is an oscillation here as well, like in membranes, between the ISO and the ΔT difference spectra both in the intensity (panel A) and the frequency (panel B) of the disappearing amide II frequency, but this oscillation disappears at the maximal H/D exchange rate (the exchange rates became so small that the frequencies could not be determined any more). In lysozyme, H/D exchange is not leading to protein heat-denaturing. Here, H/D exchange involves only reversible protein movements, the irreversible denaturing happens only at much higher temperatures.

Since earlier no such 'step' experiments were carried out to study H/D exchange, we checked whether the observed oscillation between the ISO and ΔT spectra is due to the biological membrane, or proteins alone can also exhibit similar phenomena. Therefore, we have studied, among the same conditions as above, the H/D exchange in lysozyme dissolved in the same D_2_O-based phosphate buffer as used for the membranes. The temperature dependences of the intensity and the frequency of the disappearing amide II band are shown in Figure 5.

The H/D exchange rate (the total exchange is shown in Figure 1) becomes measurable around 20°C, from where it gradually increases until about 50°C and then it drops rapidly to zero (Figure 5A). There is an oscillation between the ISO and ΔT spectra in the range of 20–50°C, but there is no correlation between H/D exchange and protein denaturing. The dynamics of the protein allows complete H/D exchange (45–55°C) before heat-denaturing is happening (70–80°C). Protein internal movements are large enough to facilitate H/D exchange, but remain in the reversible range. This is a clear difference as compared with any membrane protein studied above. The temperatures of the maximal H/D exchange rates and that of denaturing agree well with those found for lysozyme earlier in conventional H/D exchange experiments [32,33]. Similar temperature difference between completed H/D exchange and thermal denaturing has been found e.g. in cytochrome oxidase as well [34].

The frequencies of the disappearing amide II band could be determined only between 20–50°C (Figure 5B) since at lower and at higher temperatures the intensities of the difference spectra were too small. The frequencies oscillate between the ISO and ΔT points, but this oscillation diminishes between 40–50°C, where H/D exchange is the largest.

## 4. Discussion

For the first time, three parameters, H/D exchange, lipid disorder, and protein heat-denaturing were combined to describe the dynamics and the interaction between lipids and proteins in biomembranes. H/D exchange is directly measuring protein dynamics, and indirectly, via the interaction of proteins with their environment, membrane dynamics as well. Lipid disorder as measured by the ν_sym_CH_2 _frequency is also a dynamics-related parameter. In general, higher lipid disorder means higher lipid dynamics, but for example highly disordered lipids on protein-lipid interfaces do not necessarily have high dynamics.

In the membranes, the rates of the disappearance of the amide II band (Figure 3) have separated into two sets, to the lower values of the ISO and to the higher values of the ΔT difference spectra. H/D exchange rates started to increase at 30–40°C. The highest exchange rates coincided with the large-scale protein denaturing. Then, the rate decreased (in mitochondria ceased completely) as protein denaturing progressed. From this data, it seems that denaturing of membrane proteins is a consequence of events connected to H/D exchange.

In solution, as shown for lysozyme, H/D exchange was completed before protein heat-denaturing appeared. The fact that the temperature of the maximal H/D exchange rate in lysozyme (48–50°C) (Figure 5A) agrees rather well with the temperatures of the maximal exchange rates of the investigated membrane proteins (Figures 3A, C and 3E) may indicate that by their inherent nature, protein movements start roughly at the same temperatures, just the consequences of the structural fluctuations are different in solution and in the membranes. This idea is supported by early H/D exchange studies on lysozyme powder, which lead to the conclusion that 'the internal protein dynamics are not strongly coupled to surface properties' [35].

For membrane proteins, we think that the evolution of H/D exchange into protein heat-denaturing is due to the presence of lipids. The dynamics of the membrane proteins should necessarily be different from that of water-soluble ones; in a membrane, a large fraction of the protein surface is in contact with hydrophobic lipid fatty acyl chains; in water, the whole protein surface is covered with polar groups. Thus, it might be that the non-polar membrane protein surface segments can move more easily to positions from where they cannot find back to their native positions and/or even the smallest movement of the protein in the membrane is counteracted by lipid rearrangements, which make the return to the native position impossible.

If this latter was the case the highest H/D exchange rates should correlate with the lipid properties (disorder, unsaturation). But, the temperature of the maximal H/D exchange rates did not correlate either with lipid unsaturation or with lipid disorder as measured by FTIR spectroscopy. In this study, lipid fatty acyl chain disorder was characterised by the frequency of the ν_sym_CH_2 _vibration. This frequency is lower for the ordered CH_2 _segments, and higher for the disordered CH_2 _segments (which are situated more toward the end of the fatty acyl chain). The infrared spectrum reflects the average of the CH_2 _disorder, i.e. the ν_sym_CH_2 _frequency is a trans-membrane order parameter. This lipid disorder parameter is 'sensing' H/D exchange/protein denaturing when the protein-to-lipid ratio is high (e.g. in tobacco, Figure 3A), otherwise it is insensitive to changes in the protein part of the membrane (Figures 3C and 3E). The reason for this phenomenon might be that at lower protein-to-lipid ratios a small part of the fatty acyl chains is in direct contact with proteins. In contrast, when the protein-to-lipid ratio in a membrane is high, a high portion of the lipids is at the lipid-protein interface, thus higher amount of lipids is forced to change its packing upon membrane protein denaturing and this is visible in the ν_sym_CH_2 _frequencies.

It is noteworthy here that protein denaturing correlated (with a certain margin) with the loss of the photosynthetic activity in the thylakoid membranes and with the lethal temperature in rat mitochondria. In tobacco, where protein denaturing started at around 48°C, the 50% loss of photosynthetic activity is around 45°C [36]; in *Synechococcus*, these values were 53°C and 43°C [37], respectively; in mitochondrial membranes protein denaturing started at around 41°C, in agreement with the lethal body temperature. The maximal H/D exchange rates in the three organisms were at 52°C, 58°C, and 48°C, respectively.

Besides the rate of the H/D exchange, there is another parameter, the frequency of the disappearing amide II band, which can report on the dynamics of the membrane proteins. The amide II frequencies at around 1545–1550 cm^-1 ^are characteristic of α-helices [38]. The amide II frequencies observed in our experiments remained in the range of the α-helices, only that they might represent slightly different ones. Here, two phenomena were observed, which should be understood: (i) Why is the frequency of the disappearing amide II band in the ΔT spectra decreasing upon passing time and/or increasing temperature up to the denaturing of the proteins, then why does it start to increase. (ii) Why is the frequency of the disappearing amide II band in the ISO difference spectra correlating with lipid phase transition (best seen in *Synechococcus*, Figure 3D) and why is it always lower than of the ΔT spectra at the same time/temperature?

We think that the monotonously decreasing amide II frequencies in the ΔT spectra – shown in Figures 3B, D and 3F – are related to inherent properties of the proteins. In the measuring scheme, we mixed two factors, temperature and time. -

The α-helices, which are on the lipid-protein interface are surrounded by a much more hydrophobic environment as compared to those helices in the interiors of the membrane proteins. Therefore, their structure, stability and dynamics might also differ in a certain extent, and this is reflected in their infrared spectra as well. – For explaining our data, it should be assumed that the α-helices on the lipid-protein interface have higher; the α-helices in the interior of the protein lower characteristic amide II frequencies. These internal helices should be reached later/at higher temperature by the H/D exchange. Hence, denaturing-induced aggregation of certain parts of the proteins should affect H/D exchange. The access to the interior of the proteins will be similarly difficult again as it was at low temperatures and/or in earlier times. Indeed, parallel to protein denaturing the frequencies of the disappearing amide II band are increasing. This may mean that H/D exchange involves again more α-helices on the lipid-protein protein interface. These α-helices may have come to the interface upon denaturing-related rearrangements of the membrane proteins. (This phenomenon is best seen for mitochondria in Figure 3F.)

As regards the correlation of the frequencies of the disappearing amide II bands with lipid disorder in the ISO difference spectra, from the above-discussed possibilities the time-effect might be involved. Note that the intensities of the ISO difference spectra are always much lower than those of the ΔT difference spectra, i.e. a smaller number of H atoms is involved in the ISO exchange. This is understandable since the temperature step gives a boost to the H/D exchange. Among ISO conditions, however, there are no temperature-induced changes either at the lipid-protein interface or in the protein itself. The self-dynamics of the proteins governs the H/D exchange, but the access of D_2_O is controlled by the physical state of the surrounding lipids at the given temperature. That might be the reason why ISO spectra correlate with lipid disorder. This phenomenon is the clearest in *Synechococcus*, where there is an almost complete gel-to-liquid crystalline lipid phase transition (Figure 3C). (Evidently, these points of the discussion need further studies to be clarified.)

## 5. Conclusion

In conclusion, the combination of three phenomena, H/D exchange, lipid disorder and protein denaturing revealed that membrane proteins are partly governed by their own dynamics and partly by the lipid environment. It seems that lipids can affect protein dynamics primarily at lower temperatures. At the high end, there was no correlation between lipid disorder/unsaturation and the temperature of the highest H/D exchange rates/protein denaturing. Thus, at high temperatures, primarily protein self-dynamics should govern the H/D exchange. However, for a large-scale exchange, large-scale access of the external D atoms is also necessary. If D atoms can rather freely reach the membrane protein surface it means that lipids do not cover those surfaces effectively any more. Thus, proteins might minimize the exposure of their hydrophobic surfaces to the polar medium by aggregating. That is the reason why H/D exchange grows into denaturing in membrane proteins.
